# Home-based light therapy for fatigue following acquired brain injury: a pilot randomized controlled trial

**DOI:** 10.1186/s12883-021-02292-8

**Published:** 2021-07-05

**Authors:** Laura J. Connolly, Shantha M. W. Rajaratnam, Jade M. Murray, Gershon Spitz, Steven W. Lockley, Jennie L. Ponsford

**Affiliations:** 1 Monash Epworth Rehabilitation Research Centre, Epworth Healthcare, Melbourne, Australia; 2 Turner Institute for Brain and Mental Health, School of Psychological Sciences, Monash University, Melbourne, Australia; 3 Division of Sleep and Circadian Disorders, Departments of Medicine and Neurology, Brigham and Women’s Hospital, Boston, USA; 4 Division of Sleep Medicine, Harvard Medical School, Boston, USA

**Keywords:** Traumatic brain injury, Stroke, Light therapy, Fatigue, Sleepiness, Sleep disturbance

## Abstract

**Background and objectives:**

Fatigue and sleep disturbance are debilitating problems following brain injury and there are no established treatments. Building on demonstrated efficacy of blue light delivered via a lightbox in reducing fatigue and daytime sleepiness after TBI, this study evaluated the efficacy of a novel *in-home* light intervention in alleviating fatigue, sleep disturbance, daytime sleepiness and depressive symptoms, and in improving psychomotor vigilance and participation in daily productive activity, following injury

**Methods:**

The impact of exposure to a dynamic light intervention (Treatment) was compared to usual lighting (Control) in a randomized within-subject, crossover trial. Outcomes were fatigue (primary outcome), daytime sleepiness, sleep disturbance, insomnia symptoms, psychomotor vigilance, mood and activity levels. Participants (*N* = 24, *M* ± *SD*_age_ = 44.3 ± 11.4) had mild-severe TBI or stroke > 3 months previously, and self-reported fatigue (Fatigue Severity Scale ≥ 4). Following 2-week baseline, participants completed each condition for 2 months in counter-balanced order, with 1-month follow-up. Treatment comprised daytime blue-enriched white light (CCT > 5000 K) and blue-depleted light (< 3000 K) 3 h prior to sleep.

**Results:**

Random-effects mixed-model analysis showed no significantly greater change in fatigue on the Brief Fatigue Inventory during Treatment, but a medium effect size of improvement (*p* = .33, *d* = -0.42). There were significantly greater decreases in sleep disturbance (*p* = .004), insomnia symptoms (*p* = .036), reaction time (*p* = .004) and improvements in productive activity (*p* = .005) at end of Treatment relative to Control, with large effect sizes (*d* > 0.80). Changes in other outcomes were non-significant.

**Conclusions:**

This pilot study provides preliminary support for *in-home* dynamic light therapy to address sleep-related symptoms in acquired brain injury.

**Trial registration:**

This trial was registered with the Australian and New Zealand Clinical Trials Registry on 13 June 2017, www.anzctr.org.au, ACTRN12617000866303.

**Supplementary Information:**

The online version contains supplementary material available at 10.1186/s12883-021-02292-8.

## Background

Fatigue and sleep disturbance are common and debilitating problems for individuals with traumatic brain injury (TBI) and stroke, termed acquired brain injury (ABI), regardless of severity, reported by 30–70% of cases [1, 2]. They impact significantly on rehabilitation, daily activities and quality of life over many years, and contribute to depression [3, 4]. Various factors are associated with fatigue following TBI and stroke, including injury-related impairments of attention and processing speed, sleep disturbance, and depression [5, 6]. Frequent sleep disturbances include excessive daytime sleepiness (EDS), hypersomnia, insomnia, reduced sleep efficiency, changes to sleep timing and sleep apnea [7–9]. Sleep may be disrupted by damage to structures involved in homeostatic regulation of sleep and wakefulness, including the hypothalamus and brain stem [10], and by secondary factors including depression and pain [11–13].

Pharmacological interventions do not provide long-term solutions to these problems, and have negative side-effects [14–17]. Ocular light exposure elicits various ‘non-visual’ circadian, neuroendocrine and neurobehavioral responses, including resetting the circadian pacemaker [18], acute alerting effects [19] and mood enhancement [20]. These effects are intensity-dependent [21] and mediated primarily by a non-rod, non-cone photoreceptor system. A subset of intrinsically photosensitive retinal ganglion cells (ipRGCs) express the short wavelength (blue) light–sensitive photopigment melanopsin (λ_max_ ~ 480 nm) and project directly to brain areas involved in non-visual alerting and circadian light responses [22]. In laboratory settings, greatest improvements in alertness or mood are observed with exposure to blue or blue-enriched light compared to other spectra [19]. Similarly, daily home-based blue light therapy can improve depressive symptoms in seasonal affective disorder [23, 24]. Given the inter-relatedness of fatigue with daytime sleepiness, lowered mood and impaired arousal and attention following brain injury [6, 25], a treatment approach such as light therapy that simultaneously addresses these symptoms appears a promising means of reducing fatigue and sleep disturbance after brain injury.

Our research group has previously conducted a randomized controlled trial of a home-based morning light intervention in a TBI group using a portable light box, and found reductions in fatigue and daytime sleepiness following blue light treatment compared to yellow light or no-treatment controls, and a non-significant trend towards reduced depression [25]. The findings of this study suggested that light therapy may be an effective treatment for fatigue and sleepiness following brain injury. The requirement to attend to a light box for 45 min within two hours of waking may be burdensome, however, and fatigue levels returned to pre-treatment levels once intervention ceased. Similar challenges have been observed in the three other trials in mild [26, 27] and severe [28] TBI utilizing short-wavelength light, which have also observed reductions in fatigue [28], daytime sleepiness [26, 27] and depressive symptoms [26], and improvements in objective sleep measures [26]. Only one study has examined light therapy in stroke patients, finding that naturalistic lighting reduced fatigue in patients undergoing inpatient rehabilitation, but there was no control condition or follow-up of patients post-discharge [29]. The alerting effects of daytime light have been shown to persist for longer-duration exposures (e.g. [30]). We therefore aimed to test the benefits of providing therapeutic lighting in the ambient environment all day and evening, reducing patient burden and potentially increasing treatment efficacy. Specifically, the study aimed to compare the impact of exposure to a dynamic light schedule with participants’ usual lighting on fatigue (primary outcome), as well as daytime sleepiness, sleep quality, insomnia symptoms, psychomotor vigilance, mood and productive activity as secondary outcomes. It was hypothesized that exposure to the therapeutic lighting would lower fatigue, daytime sleepiness and insomnia symptoms, and improve sleep quality, psychomotor vigilance, mood, activity levels, community participation levels and quality of life relative to usual lighting conditions.

## Methods

### Design

**Fig. 1:**
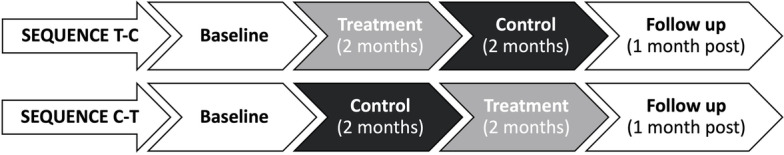
Study design showing 2 × 2 crossover sequences

A randomized within-subject, crossover trial design was employed. The protocol for each participant was 5.5 months in length, including 2-week baseline, two 2-month conditions (treatment and control), and 1-month follow-up (see Fig. 1 for study design). The study employed a cross-over design; thus all participants were exposed to both lighting conditions. There was no wash-out period between conditions, as carryover effects of the light were considered negligible, and the effect of either lighting condition was considered to be removed with the removal of the lighting [30]. Sinclair et al. [25] obtained a large effect for the primary outcome. A power analysis (G*Power [31]) undertaken with power (1-β) set at 0.80 (with α = 0.05; [32]) to detect a medium effect size (*dz* = 0.60) showed a required sample size (within-subjects) of 24.

### Participants

Participants were individuals with mild-severe TBI or stroke sustained at least 3 months earlier, living in the community. Inclusion criteria included documented history of mild-severe TBI, or stroke, and self-reported significant fatigue (Fatigue Severity Scale ≥ 4). Exclusion criteria included other medical illness causing fatigue, including other neurological disorders, pre-injury sleep disorders, including obstructive sleep apnea [33] or chronic fatigue syndrome, presence of visual impairments affecting sensitivity and response to light, transmeridian travel within preceding six weeks, current use of prescribed and over-the-counter sleep medications and inability to give informed consent as assessed by the referring clinician. Use of antidepressants was permitted (*n* = 5) provided a stable dosage was maintained throughout the study.

### Procedures

The study was approved by human research ethics committees at Epworth Healthcare (#EH2016-164) and Monash University (#9246). Participants provided written informed consent. No compensation was provided to participants. The study adheres to CONSORT guidelines.

Participants were recruited by hospital or community clinicians, from routine follow-up of people with TBI in a longitudinal outcome study and via advertisement within stroke support organizations. Interested individuals received a study explanation and eligibility screening. Following consent, injury details were obtained from medical records, including injury date, initial Glasgow Coma Scale (GCS) score, post-traumatic amnesia (PTA) duration, other injuries, MRI/CT scan results for TBI patients and for stroke patients date and nature of stroke, CT scan, treatment and medication details. Outcome measures were administered at baseline and monthly intervals (mid- and end of Treatment/Control condition), and one-month follow-up. Participants completed daily sleep and activity diaries, and wore an actigraphy device daily throughout the study.

For randomization, an independent researcher used online randomization software (www.randomization.com), based on random permuted block sizes of two and four, and transcribed allocation sequences onto cards in sealed envelopes, opened after baseline assessment by the study coordinator. Assessments were conducted by a researcher blinded to the lighting condition being received.

### Lighting intervention

Participants’ current lighting was assessed before study commencement. Priority for both Treatment and Control lighting installation was given to rooms in which participants spent most time (e.g., lounge, kitchen, bedroom, bathroom). The Colormunki Light Meter (X-Rite, Grand Rapids, MI, USA) was used to measure participants’ home lighting conditions (specific spot measurements at fixed height in vertical (54″) and horizontal (72″) planes) and data analyzed using f.luxometer software (f.lux, Los Angeles, CA, USA). We utilized recently published International Commission on Illumination (CIE) International Standard CIE S026/E:2018 to quantify the lighting [34]. The melanopsin photoreceptor predominantly mediates non-visual responses, and changing these levels was the target of the study. Equivalent Daylight (D65) Illuminance (EDI) was calculated for each photoreceptor, including melanopsin as well as the melanopic Daylight Equivalent Ratio (DER), which expresses melanopic EDI as a function of photopic illuminance (lux); higher melanopic DER values represent relatively greater melanopsin stimulation.

The active lighting intervention consisted of short-wavelength enriched high-intensity white light with correlated color temperature (CCT) of approximately > 5000 K during the day. In the evening, for 3 h prior to sleep, participants were instructed to change which lights they used to reduced intensity, short-wavelength-depleted white light (< 3000 K) provided. Participants were asked to maintain as stable as possible lighting schedule day-to-day, and light exposure was timed relative to individual sleep patterns. Lighting fixtures and lamps were selected to integrate with participants’ existing lighting arrangements. Under some circumstances, fixed spectrum lighting was used, using the concept of ‘day’ and ‘evening’ light. For example, if two circuits existed in a room, one circuit was reserved for day-time high intensity blue-enriched light (e.g. ceiling light) and another for evening and night-time with dimmer blue-depleted light (e.g. table lamp). If this was not possible, table and bedside lamps were provided for evening use. Tunable and programmable lamps were programmed to change lighting automatically at the right time of day (Smart Wi-FI LED Bulb, TP-Link, Shenzhen, China; Genesis DynaSpectrum HealthE LED Lamp, Lighting Science, RI, USA). Participants were given written and verbal instructions on use and timing of lights for each condition. In the sham control condition, lamps were changed as per Treatment condition but did not change in color temperature or intensity from participants’ normal lighting (typically ~ 3000-4000 K). All lighting was commercially available and within safety standards for residential lighting. A qualified electrician changed light fittings or bulbs in participants’ homes. At each monthly visit, participants were asked to reflect upon their compliance with treatment lighting when at home, and transitioning from day to nighttime at the designated hour. Further details of the lighting protocol are documented in a separate paper, with two case studies illustrating our approach (Connolly et al., submitted).

### Measures

**Baseline and Screening Measures** included the following:

**Demographics questionnaire:** age, gender, educational history, occupational history, ethnicity, living circumstances and whether the individual had a bed partner.

**Medical records:** date of injury, initial GCS, duration of PTA, other injuries, MRI/CT scan results, date and nature of stroke, treatment and medication details.

### Outcome measures

All measures had been previously used in TBI and stroke populations. The *primary outcome* measure was the **Brief Fatigue Inventory (BFI)** [35], a 9-item scale used to capture current fatigue levels on a scale of 0 (no fatigue or does not currently interfere) to 10 (bad fatigue that completely interferes with activity/work) in the previous 24 h (state fatigue).

*Secondary Outcomes* included the following measures.

**Fatigue Severity Scale (FSS)** [36] is a 9-item self-report measure assessing impact of fatigue on daily activities or trait-like fatigue, on a 7-point scale from 1 (strongly disagree) to 7 (strongly agree). A mean item score ≥ 4 indicates clinically significant fatigue.

**Epworth Sleepiness Scale (ESS)** includes 8 items assessing a person’s likelihood of falling asleep during everyday activities such as “Watching TV” or “Sitting quietly after a lunch without alcohol” [37]. Score > 10 suggests clinically significant daytime sleepiness [37].

**Pittsburgh Sleep Quality Index (PSQI)** assesses subjective global sleep quality [38] in past month (e.g. bedtime, sleep duration) and frequency of problems interfering with sleep. Lower scores indicate greater sleep quality. Scores ≥ 8 indicate clinical insomnia [39].

**Insomnia Severity Index (ISI)** [40] screens for insomnia with 7 questions rated on a 5-point scale, (0 = no problem, 4 = very severe problem). A score of 8–14 indicates subthreshold insomnia, 15–21 clinically moderate, and ≥ 22 severe clinical insomnia.

**Psychomotor Vigilance Task (PVT*)*** (10 min) measured reaction time (MATLAB v. R2018b) once between 10am-5 pm at each of the assessment timepoints [41]. Prior research has demonstrated exposure to short wavelength light, decreases reaction time and errors on this task [42].

**Hospital Anxiety and Depression Scale (HADS)** measured self-reported depression symptoms [43] with the depression (HADS-D) subscale. The 14 items are rated on a 4-point scale, where 0 = “Not at all” and 3 = “Most of the time”.

**Participation Objective Participation Subjective (POPS **[44]**)** assessed community, work and social participation, with higher scores indicating greater participation.

**Activity diary**. A customized activity questionnaire was completed daily at 9 pm and used to calculate number of minutes spent on activity, rest and sleep in four time-blocks (between 9am and 9 pm). Activity encompassed physical and mental activity (e.g. doing chores, reading), rest was “giving the body a break” (e.g. lying down, listening to music) and sleep included napping. Percentage of daily productive activity was calculated as time spent on physical and mental activity relative to rest or sleep.

**Side Effects Questionnaire** was used to capture side effects experienced, including headache, nausea, cognitive changes, and appetite, at each assessment.

**End of Light Therapy Questionnaire** was completed at follow-up, to capture participants’ qualitative experiences of the lighting interventions and subjective symptoms.

**Actigraphy and sleep diary**. Participants recorded sleep and wake times and other sleep phenomena, in a daily sleep diary throughout the study, including time to fall asleep, awakenings after sleep onset, and daytime naps. They also wore wrist actigraphy devices (Actiwatch-2, Actiwatch Spectrum or Actiwatch Spectrum Plus; Philips Respironics, Bend, OR, USA) on the non-dominant wrist, with activity measured in 1-min epoch as sleep or wake. Actigraphic sleep parameters were analysed for sleep episodes identified in the sleep diaries.

### Data analysis

**Fig. 2:**
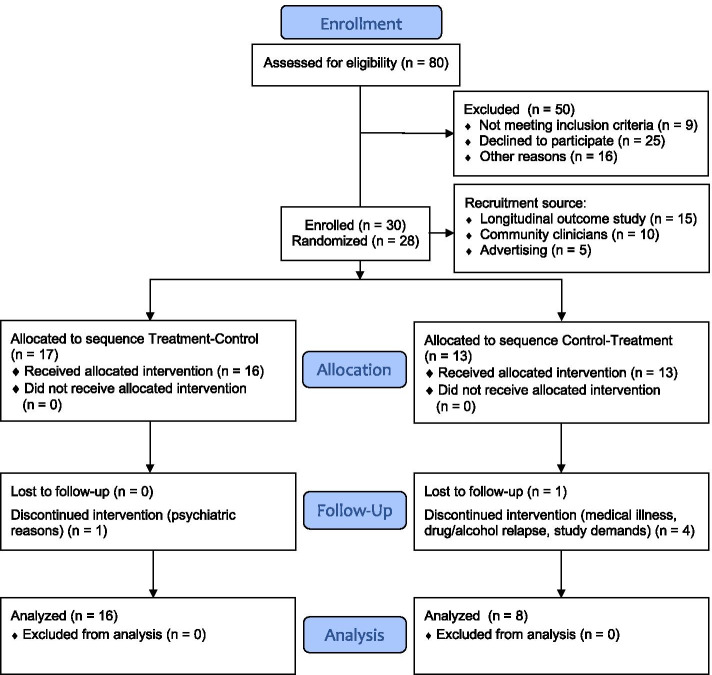
CONSORT flow diagram of recruitment and participant retention. Note: The first two participants were not randomized as we were initially examining the feasibility and acceptability of the methods. No changes were made to the protocol so these cases were included

Thirty participants were enrolled and 28 randomized, with 24 completing the study and included for analysis (see Fig. 2 CONSORT chart). All variables met assumptions of linearity, homogeneity of variance and had normally distributed residuals. A linear mixed-model analysis was used to model each outcome variable as a linear function of treatment (i.e., end treatment or end control), period (i.e., differences between condition 1 and condition 2 for treatment–control and control-treatment) and sequence (i.e., participants allocated treatment–control vs. participants allocated control-treatment), with participant included as a random variable. The analysis controlled for baseline scores and injury type (TBI and stroke). Random effects were included for participants intercepts. The primary indicator of a treatment effect was interaction of time by treatment group. Results were considered significant if two-tailed *p* value was < 0.05. Data analysis was performed using RStudio [45] and *lme4* [46].

### Actigraphy data analysis

Individual actigraphic sleep episodes were inspected and aligned with sleep diaries by inputting of subjective sleep and wake times by an independent researcher blinded to study conditions. When discrepancies ≥ 60 min between actigraphy data and sleep diary entries were identified, the following approach was used [47]: If subjective bedtime was reported as ≥ 60 min before a substantial reduction in activity and light levels, bedtime was adjusted to the time of decreased activity and light; if reported wake time was ≥ 60 min after a substantial increase in activity and light, wake time was shifted to the start of the sustained activity and light increase. Actigraphy data was excluded from analysis in cases of equipment malfunction or insufficient data to determine night time sleep episodes. In total, there were 663 (17%) nights missing (total of 3976) across all study periods; 155 (12%) missing from Treatment, and 171 (15%) from Control.

The following six outcomes were derived, averaged across each study condition for each participant: total sleep time (TST), sleep onset latency (SOL), wake after sleep onset (WASO), sleep efficiency (%), and participants’ average sleep and wake times. Actigraphy-derived sleep parameters obtained during baseline and mid to end condition periods were utilised in linear mixed-model analyses.

## Results

### Participant characteristics

Of 80 individuals screened for the study, 30 were enrolled and 28 randomized. The first 2 participants were allocated to treatment–control sequence but standard study protocols and blinded follow-up were applied. At screening, all participants reported clinically significant fatigue (FSS ≥ 4). During the study, 6 participants withdrew, 2 during baseline, 4 during condition 1 (3 control, 1 treatment). Reasons for withdrawal included drug and alcohol relapse (*n* = 1), research demands (*n* = 2), psychiatric/medical illness (*n* = 2) and lost to follow-up (*n* = 1). Figure 2 shows the study CONSORT chart. Participants completed the study year-round in Melbourne, Australia.

Table 1 shows sample demographic and clinical characteristics by treatment sequence. A summary of participant demographics can be found in Supplementary Table 1. Time since injury ranged from 1 to 26 years (*M* = 10.23, *SD* = 9.34). Causes of injury included motor vehicle accident (21%), stroke (21%), motorcycle (8%) and bicycle (12.5%) accidents, pedestrian accident involving motor vehicles (12.5%), falls (4%), sporting injury (8%) and other causes (8%). Duration of PTA was on average 24 days (SD = 25 days, range = 12 h-80 days). Based on PTA, most participants had severe injury (PTA > 1 week, 53%), followed by moderate (PTA 1 – 7 days, 26%), and mild (PTA < 24 h, 17%). Five participants had stroke (2 ischemic, 3 unknown). Average time since stroke was 9 years. Participants in each sequence did not significantly differ on outcome measures at baseline.

**Table 1:** Demographic and clinical characteristics by treatment sequence at baseline^a^

Baseline variables	Treatment-placebo (*n* = 16)		Placebo-treatment sequence (*n* = 8)		Total (*N* = 24)	
	*M*	*SD*	*M*	*SD*	*M*	*SD*
Age (years)	43.13	10.67	46.75	13.13	44.33	11.39
Gender (female)	7 (43.75%)		3 (37.50%)		10 (41.66%)	
Injury type (TBI)	14 (87.50%)		5 (62.50%)		19 (79.17%)	
BFI	5.33	1.75	6.75	0.85	5.81	1.64
FSS	5.07	1.27	5.85	0.64	5.33	1.15
ESS	7.69	3.89	10.25	5.20	8.54	4.43
PSQI	7.81	4.09	9.38	4.03	8.33	4.05
ISI	13.19	5.72	13.25	6.80	13.21	5.95
HADS (Depression)	6.31	3.40	8.25	3.01	6.96	3.34
PVT: mean RT^b^	345.82	89.11	332.68	62.96	321.58	52.41
PVT: Fastest 10% RT^b^	255.07	41.58	252.63	28.69	245.50	27.32
POPS	-0.12	0.53	-0.50	0.44	-0.24	0.52
Productive Activity (%)	85.82	12.28	84.17	9.67	85.16	10.96
Sleep onset^c,d^	22.90	0.87	23.62	0.98	23.13	0.95
Sleep offset^c,d^	30.95	0.64	31.12	1.05	31.00	0.77
SOL^c^	16.40	12.66	21.19	12.66	17.91	12.52
WASO^c^	45.38	20.32	40.25	13.18	43.76	18.15
TST^c^	437.29	31.73	426.73	66.72	433.96	43.97
Sleep efficiency (%)^c^	86.40	4.67	85.67	5.06	86.17	4.66

*Notes.* Baseline data is for participants who completed the study and were included in analyses

*Abbreviations*: *BFI* Brief Fatigue Inventory, *ESS* Epworth Sleepiness Scale *FSS* Fatigue Severity Scale, *HADS* Hospital Anxiety and Depression Scale, *ISI* Insomnia Severity Index, *POPS* Participation Objective Participation Subjective (Objective Subscale only), *PSQI* Pittsburgh Sleep Quality Index, *PVT* Psychomotor Vigilance Task, *SOL* Sleep onset latency, *TST* Total sleep time, *WASO* Wake after sleep onset

^a^Data are mean (*M*) and standard deviation (*SD*), or percentage values, of participant demographics and baseline characteristics shown for each treatment group

^b^*N* = 18; results not available for some participants due to equipment failure

^c^*N* = 19; results not available for some participants due to equipment failure

^d^Clock times were converted to 24 h time with decimals. Sleep offset times were adjusted + 12 to reflect a later time than sleep onset numerically

### Light measurement

Table 2 shows average photopic lux, irradiance and CCT values, plus α-opic EDI, and melanopic DER values across study conditions and rooms, measured in the horizontal plane at a height of 72”.

**Table 2:** Control and treatment condition lighting measurements

Location	Time of day	*N*	Photopic lux Mean ± SD	CCT (K) Mean ± SD	Irradiance uW/cm^2^ Mean ± SD	S-cone-opic a-opic EDI (lux) Mean ± SD	M-cone-opic a-opic EDI (lux) Mean ± SD	L-cone-opic a-opic EDI (lux) Mean ± SD	Rhodopic a-opic EDI (lux) Mean ± SD	Melanopic a-opic EDI (lux) Mean ± SD	Melanopic DER Mean ± SD
**Control lighting**											
Bedroom	Day	24	338.06 ± 269.96	3539 ± 861	106.87 ± 100.27	165.68 ± 134.47	290.39 ± 230.74	340.31 ± 271.31	223.12 ± 176.15	201.69 ± 159.17	0.57 ± 0.13
	Evening	22	251.86 ± 175.24	3297 ± 946	70.19 ± 62.77	105.81 ± 100.42	209.51 ± 150.92	254.02 ± 175.99	150.26 ± 117.87	131.79 ± 108.46	0.50 ± 0.13
Bathroom	Day	18	490.07 ± 488.26	3660 ± 978	171.62 ± 251.86	214.99 ± 206.73	410.30 ± 391.30	494.63 ± 497.66	302.57 ± 282.42	267.97 ± 250.35	0.57 ± 0.13
	Evening	8	526.13 ± 271.62	3615 ± 1162	160.96 ± 83.85	239.40 ± 159.44	441.69 ± 227.45	528.46 ± 273.61	318.22 ± 170.36	278.33 ± 153.57	0.53 ± 0.15
Living	Day	23	329.68 ± 252.36	3255 ± 4501	99.73 ± 92.50	135.44 ± 111.39	274.50 ± 209.58	332.98 ± 254.97	199.26 ± 154.86	175.37 ± 138.60	0.53 ± 0.09
	Evening	20	211.23 ± 150.79	2988 ± 360	62.58 ± 49.48	73.10 ± 58.80	170.00 ± 121.29	213.93 ± 152.57	114.27 ± 82.48	97.10 ± 71.46	0.47 ± 0.08
Dining	Day	16	441.66 ± 328.29	3532 ± 751	138.39 ± 125.58	223.66 ± 176.40	367.88 ± 286.16	440.28 ± 331.79	284.34 ± 216.55	267.66 ± 194.53	0.58 ± 0.13
	Evening	12	350.06 ± 232.39	3402 ± 1002	107.14 ± 77.03	155.92 ± 146.97	292.40 ± 204.06	351.86 ± 229.13	209.35 ± 153.78	181.89 ± 133.46	0.52 ± 0.13
Kitchen	Day	21	462.09 ± 321.32	3507 ± 653	136.49 ± 127.84	231.27 ± 193.02	397.13 ± 284.48	464.98 ± 322.92	304.77 ± 233.42	275.71 ± 217.17	0.57 ± 0.09
	Evening	14	327.66 ± 197.03	3369 ± 770	90.99 ± 72.66	157.89 ± 137.04	278.18 ± 177.87	329.32 ± 196.21	206.32 ± 147.27	183.85 ± 137.21	0.52 ± 0.10
Study	Day	10	322.66 ± 319.21	3267 ± 544	92.96 ± 137.85	152.28 ± 199.59	275.83 ± 292.53	325.33 ± 319.75	210.98 ± 253.61	190.39 ± 239.65	0.53 ± 0.11
	Evening	8	190.25 ± 99.93	2991 ± 403	44.57 ± 35.31	68.95 ± 40.91	153.95 ± 81.89	192.72 ± 101.04	104.72 ± 57.47	89.81 ± 49.97	0.46 ± 0.08
**Treatment lighting**											
Bedroom	Day	19	329.20 ± 311.48	5219 ± 527	92.73 ± 109.31	261.67 ± 253.73	311.77 ± 294.11	327.36 ± 310.04	279.28 ± 263.85	268.43 ± 254.35	0.82 ± 0.09
	Evening	20	195.40 ± 173.56	2685 ± 365	53.52 ± 52.50	56.94 ± 59.21	153.28 ± 137.97	198.43 ± 175.63	97.79 ± 89.93	80.16 ± 74.37	0.39 ± 0.08
Bathroom	Day	16	482.24 ± 351.35	4984 ± 897	140.04 ± 123.63	358.95 ± 280.30	447.88 ± 326.62	479.65 ± 349.97	387.03 ± 285.93	366.03 ± 273.53	0.75 ± 0.12
	Evening	6	321.87 ± 225.76	2785 ± 183	88.91 ± 75.97	92.81 ± 62.84	252.55 ± 174.79	325.97 ± 228.96	158.34 ± 107.19	127.51 ± 85.04	0.40 ± 0.03
Living	Day	19	310.65 ± 179.67	5434 ± 588	89.74 ± 73.36	260.87 ± 163.21	295.11 ± 172.71	308.50 ± 178.30	264.92 ± 158.66	255.27 ± 154.36	0.81 ± 0.06
	Evening	18	157.42 ± 180.62	2910 ± 517	46.74 ± 57.41	52.91 ± 65.12	126.45 ± 146.31	159.20 ± 182.33	83.60 ± 97.86	70.00 ± 82.32	0.44 ± 0.08
Dining	Day	15	573.92 ± 498.79	5252 ± 1049	150.70 ± 147.44	479.79 ± 449.44	542.86 ± 477.67	571.38 ± 496.10	487.74 ± 439.60	470.56 ± 428.79	0.79 ± 0.12
	Evening	9	473.78 ± 390.72	2947 ± 196	126.16 ± 124.14	164.18 ± 137.06	383.14 ± 316.41	478.48 ± 394.27	255.46 ± 210.78	214.69 ± 176.66	0.44 ± 0.04
Kitchen	Day	20	518.73 ± 432.48	5179 ± 963	163.82 ± 171.25	420.19 ± 386.29	488.28 ± 418.67	516.28 ± 429.39	434.37 ± 389.77	416.71 ± 379.94	0.78 ± 0.13
	Evening	8	336.85 ± 213.52	3050 ± 214.92	90.62 ± 76.69	118.48 ± 70.14	272.25 ± 168.66	340.09 ± 215.32	182.48 ± 108.11	153.06 ± 87.90	0.46 ± 0.06
Study	Day	9	385.50 ± 438.61	5586 ± 1001	121.02 ± 191.54	331.67 ± 367.51	366.42 ± 415.20	384.23 ± 438.07	333.98 ± 379.92	323.71 ± 367.71	0.82 ± 0.13
	Evening	6	92.57 ± 80.97	2835 ± 584	22.67 ± 25.60	27.76 ± 27.26	73.29 ± 65.34	93.88 ± 81.68	47.40 ± 42.85	39.20 ± 35.55	0.43 ± 0.12

The aim of the intervention was to increase the melEDI and DER during the day and decrease them during the evening during the Treatment compared to the Control intervention (final two columns). This was typically associated with a daytime increase and evening decrease in CCT during the Treatment

*Notes*. *Abbreviations*: *CCT* correlated color temperature, *EDI* Equivalent Daylight Illuminance, *DER* Daylight Equivalent Ratio. Only the melDER is shown here; DER values for the other photoreceptors can be calculated by dividing the α-opic EDI value by the photopic lux provided

Paired-samples t-tests showed that there was no significant difference in photopic lux between Treatment (441 ± 267 lx) and Control (394 ± 216 lx) during the daytime (*p* = 0.28) but, as intended, the installation of blue-enriched lamps with significantly higher CCTs during Treatment (~ 5200 K versus 3500 K), significantly increased melanopic illuminance by ~ 55%, from 226 ± 143 melEDI lux during the Control to 350 ± 225 melEDI lux during the Treatment, with a corresponding increase in the melanopic DER (Control 0.56 ± 0.07, Treatment 0.80 ± 0.07) (Table 2; Supplementary Table 3; all *p* < 0.05).

For evening settings, there was a significant reduction in photopic illuminance during the Treatment compared to Control condition in the evening (210 ± 152 lx versus 272 ± 132 lx, respectively; *p* = 0.009). Combined with the significant reduction in CCT during Treatment (2865 K versus 3214 K), there was a ~ 20% overall reduction in melanopic illuminance, from 139 ± 77 melEDI lux during Control to 96 ± 65 melEDI lux during Treatment, with a corresponding decrease in the melanopic DER (Control 0.49 ± 0.07, Treatment 0.42 ± 0.07) (all *p* ≤ 0.001).

### Treatment compliance and qualitative feedback

Participants reported average compliance rates of 81% during the treatment phase, although three participants with self-reported memory complaints expressed difficulty recalling their performance across time.

In the End of Light Therapy Questionnaire, most participants reported positive experiences during the study in terms of their symptoms; seventeen participants were “mostly satisfied” or “very satisfied” with the treatment, and three individuals reported “some satisfaction”. Nineteen out of twenty-three (one no response) reported a preference for incorporating light therapy in their day-to-day life following the cessation of the study. Seventy-one percent of responders had a preference for the Treatment condition lighting over Control lighting.

### Outcome measures

**Fig. 3:**
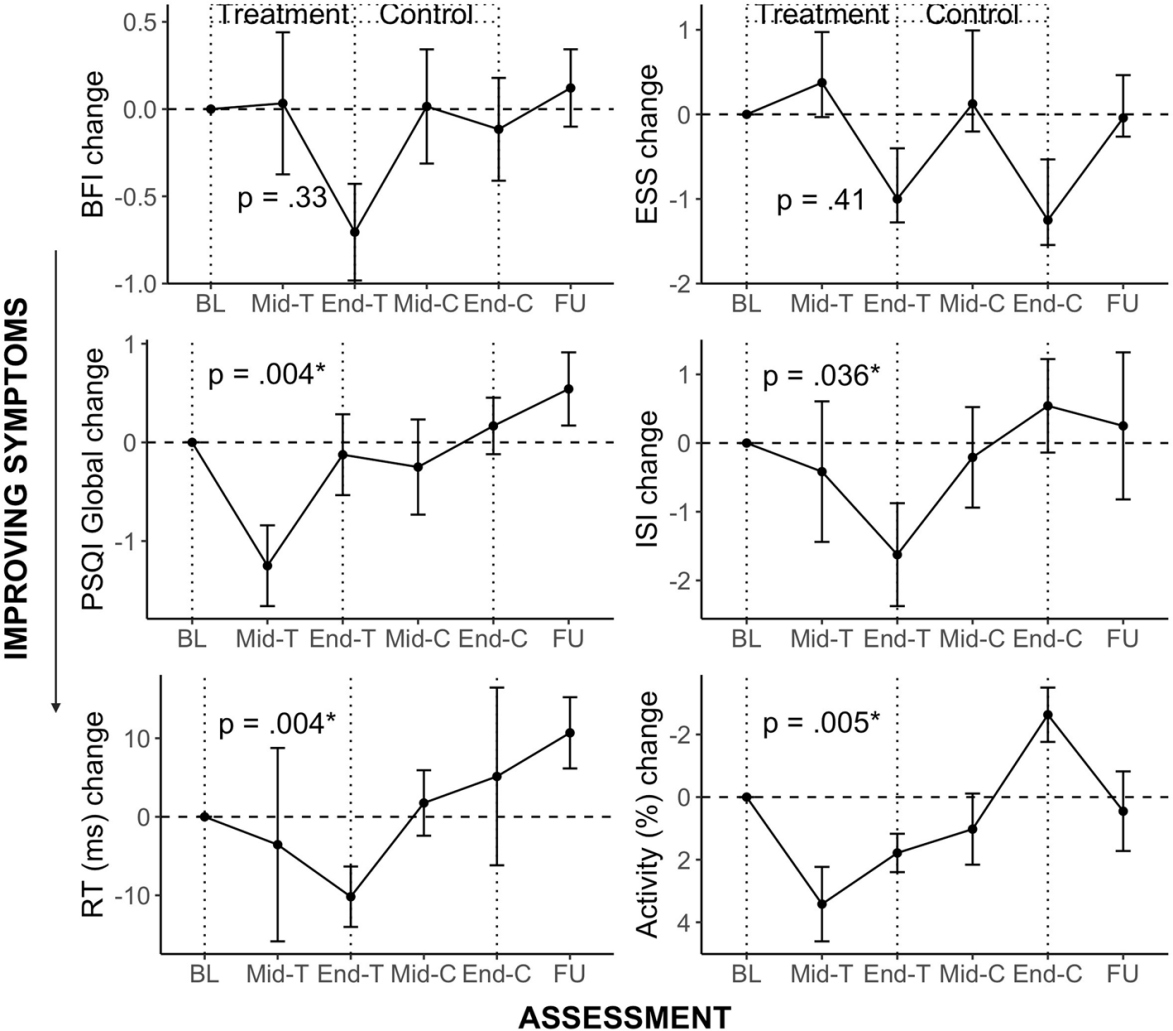
Change in fatigue (BFI), daytime sleepiness (ESS), sleep quality (PSQI), insomnia symptoms (ISI), mean reaction time (ms), and productive activity (%) across study time points. Notes. Condition sequences are combined in the above figures. Error bars represent standard error, BL = baseline, T = Treatment, C = Control, FU = follow up

Figure 3 shows the change in symptom severity for primary and secondary outcomes at baseline, mid- and end-treatment and end-control, and follow-up.

Table 3 summarizes results of linear mixed-model analyses on primary and secondary outcomes, and descriptive statistics of the end Treatment assessment. Treatment was not associated with statistically significant changes in the primary outcome of fatigue (BFI) relative to placebo, although there were trends for improvement with medium effect size (*p* = 0.33, *d* = -0.42). Furthermore, changes on the secondary outcomes of fatigue (FSS) (*p* = 0.20, *d* = -0.55) and daytime sleepiness (ESS) (*p* = 0.41, *d* = 0.35) were not statistically significant, but also showed medium effect sizes. It should be acknowledged, however, that a decrease in daytime sleepiness was also observed at the end of Control assessment point, a pattern not observed with other variables. Treatment was, however, associated with a significant improvement in measures of subjective sleep; with significant reductions in sleep disturbance (PSQI) by 1.50 points on average and insomnia symptoms (ISI) by 2.13 points, relative to baseline. There were no statistically significant changes in actigraphy-derived sleep during treatment.

**Table 3:** Mixed-model results, including treatment estimate, effect size and significance level

Outcome	*n*	*M/SE*	Treatment effect estimate (95% CI)	*t* stat	Effect size (*d)*	*p* value
BFI	24	4.88 (0.43)	-0.39 (-1.16, 0.39)	-0.97	-0.42	.33
ESS	24	7.00 (0.80)	0.59 (-0.83, 2.01)	0.82	0.35	.41
FSS	24	5.12 (0.25)	-0.29 (-0.73, 0.15)	-1.29	-0.55	.20
HADS (Depression)	24	6.42 (0.78)	-0.69 (-1.95, 0.58)	-1.07	-0.45	.29
ISI	24	11.08 (1.12)	-2.06 (-3.99, -0.14)	-2.06	-0.89	**.036**
PSQI	24	6.83 (0.74)	-0.97 (-1.62, -0.32)	-2.92	-1.24	**.004**
Mean RT	18	300.22 (7.10)	-28.36 (-47.48, -9.23)	-2.91	-1.75	**.004**
Fastest 10% RT	18	234.20 (4.98)	-15.10 (-25.03, -5.16)	-2.98	-2.98	**.003**
POPS	24	-0.34 (0.10)	-0.07 (-0.21, 0.06)	-1.04	-0.32	.30
Activity Diary	15	90.54 (2.06)	2.95 (0.91, 4.98)	2.83	1.63	**.005**
Sleep onset	19	22.95 (0.34)	-0.12 (-0.80, 0.57)	-0.34	-0.17	.74
Sleep offset	19	29.86 (0.53)	0.39 (-0.70, 1.47)	0.79	0.36	.48
Sleep onset latency (SOL)	19	18.46 (2.78)	0.47 (-4.75, 5.69)	0.18	0.10	.86
Wake after sleep onset (WASO)	19	46.51 (5.97)	-0.54 (-10.73, 9.65)	-0.10	-0.05	.92
Total sleep time (TST)	19	400.23 (17.98)	-12.87 (-42.22, 16.47)	-0.86	-0.49	.39
Sleep efficiency	19	82.23 (1.72)	-0.34 (-3.83, 3.16)	-0.19	-0.09	.85

*Notes. M/SE* calculated from end Treatment assessment

*Abbreviations*: *BFI* Brief Fatigue Inventory, *ESS* Epworth Sleepiness Scale, *FSS* Fatigue Severity Scale, *HADS* Hospital Anxiety and Depression Scale, *ISI* Insomnia Severity Index, *POPS* Participation Objective Participation Subjective (Objective Subscale only), *PSQI* Pittsburgh Sleep Quality Index, *PVT* Psychomotor Vigilance Task, *SOL* Sleep onset latency, *TST* Total sleep time, *WASO* Wake after sleep onset

On behavioral outcomes; there was a significant reduction in mean RT and fastest 10% RT on a measure of psychomotor vigilance, both with large effect (*d* > 0.80). There was also a significant increase in daily reported productive activity (*d* > 0.80), increasing by an average of 5.40% from baseline to end-treatment. There were no significant differences in subjectively rated levels of participation (POPS) or depressive symptoms (HADS), however. Treatment sequence was not significant across primary and secondary outcomes measures.

### Side-effects

A summary of reported side-effects can be found in Supplementary Table 2. Reported side-effect symptom severity/frequency did not differ significantly across treatment conditions. The most commonly reported side effects across both conditions were headache (mild-severe), eye irritation (mild), sleep problems (mild-moderate), thought/concentration problems (mild-moderate), drowsiness (mild-moderate), fatigue (mild-severe) and mood changes including irritability and feeling depressed (mild-moderate). All symptoms resolved spontaneously and did not result in study withdrawal. When reporting symptoms, participants cited as reasons for symptoms; their brain injury, increased work commitments, psychosocial stressors, or a new injury or illness. Two participants reported mild eye sensitivity when commencing the treatment lighting intervention which they thought may have been related to the lights; however in discussion with study researchers, they expressed no desire for modifications or study discontinuation.

## Discussion

The aim of the current pilot study was to investigate the efficacy of a dynamic home-based light therapy in reducing fatigue and sleep disturbance in individuals with TBI and stroke. Treatment was not associated with statistically significant change on the primary outcome measure of fatigue, although there was a medium effect size of improvement found. We did, however, observe a significant reduction in subjective sleep quality, specifically sleep disturbance and insomnia symptoms. We also observed significant improvements across behavioral measures during the treatment phase, in psychomotor vigilance (mean RT and fastest 10% RT), and percentage of time in productive activity. There were no significant changes in daytime sleepiness, actigraphy-derived sleep measures nor depressive symptoms or community and social participation levels. Our study did not find a significant effect of treatment sequence or injury type (TBI v stroke), although the current pilot only included five stroke participants. Our findings suggest that light therapy may be useful in treating sleep disturbance, increasing speed of information processing and improving productive activity in individuals with TBI and stroke.

Our findings contrast with those of prior studies which examined time-limited exposure to blue or bright light LED devices in those with TBI [25, 26, 28] or cancer [48, 49], and found significant reductions in fatigue and daytime sleepiness. One potential reason for this difference is the nature of the lighting employed. These previous studies utilized LED devices at close proximity during mornings which may be more likely to exert an impact on an individual’s daytime fatigue and sleepiness. Further, compared to studies which did modify ambient lighting with inpatients [50] and office workers [51], our intervention utilized existing home fixtures which are typically lower in illuminance than hospital and office settings (when measured at a comparable distance from the source), and may have resulted in lower effectiveness. To our knowledge, this is the first home-based light therapy in individuals with ABI utilizing participants’ existing light fixtures. We did find, as intended, that on average Treatment lighting had significantly greater melanopic illuminance during daytime, and significantly less melanopic illuminance during evenings, relative to Control lighting. Despite the non-significant results for fatigue, trends in the anticipated direction were observed, and other sleep-related outcomes did show significant changes. The small sample size likely reduced the available power and therefore further analysis with a larger sample and better monitoring of exposure compliance is desirable.

Our study found a significant reduction in subjective sleep disturbance and insomnia symptoms during dynamic light treatment where previous studies did not. Studies in TBI [25, 27, 28] patients utilizing time-limited morning exposure to devices, including that of Sinclair et al., and in stroke patients [29], using naturalistic light, found no significant reduction in sleep disturbance. This was supported by a recent network meta-analysis which suggested that blue-wavelength light therapy did not significantly reduce sleep disturbance [52]. This difference might be explained by the lack of exposure to night-time intervention lighting in these prior studies, whereas we significantly reduced both the illuminance and short-wavelength content of evening exposure in the current study. Exposure to blue-enriched evening light has been shown to increase evening alertness and negatively impact subsequent sleep [53–55]. Evening light exposure is a necessary consideration in future studies. A recent study of indoor lighting showed that half of homes have lighting sufficient to suppress melatonin by 50%, as pre-bed melanopic illuminance is typically too great in evenings, which by consequence disrupts sleep [56]. It is therefore plausible that using a dynamic 24-h interventional lighting system would be more likely to modify participants’ sleep than bright light or single-spectrum light exposure employed only during mornings. Our study findings of a reduction in sleep disturbance speaks to the strength of a more comprehensive ambient lighting intervention.

Only two previous studies investigating morning light therapy after ABI have utilized objective sleep measures, and results have been mixed. One study found that blue light produced significant phase advances in sleep onset relative to amber light, but no changes in other actigraphy-derived sleep parameters [27]. Another found an increase in total sleep time and sleep efficiency, and a decrease in wake after sleep onset (WASO), in the blue-light treatment group compared with amber light [26]. It is noted, however, that participants in these studies had mild injury and were < 10 months post-injury, and the opportunity for improvement in sleep may have been greater than in the current sample (on average 10 years post-injury).

We observed a significant reduction in psychomotor reaction time during the dynamic light treatment compared to the control light. While two previous light trials in TBI were unable to find significant improvements in psychomotor vigilance [25, 27], our results mirror those observed in individuals with mild TBI who exhibited reduced reaction time following exposure to blue light therapy [57], and in healthy individuals, who demonstrated improvements in psychomotor vigilance tasks after exposure to short-wavelength light [19, 30, 58]. The current study also showed a reported average increase of ~ 5% in productive activity from baseline to end of treatment. No previous trials have examined the impact of light therapy on activity in TBI or stroke, although increased actigraphy-assessed average physical activity count (+ 18% from baseline) has been observed following daily bright light therapy in Parkinson’s Disease [59]. While modest, these results are encouraging and suggest that a home-based dynamic light therapy may improve daytime functioning in individuals with brain injury.

The pattern of symptom change observed suggests that a mild reduction in symptoms occurred during the first 4 weeks of the 8-week treatment phase, with most positive gains occurring in weeks 4–8 of Treatment (e.g. BFI, ESS, ISI, RT, Fig. 3). This finding contrasts with prior controlled exposure studies [30] and field studies in schools [60] that describe an immediate impact of light on EEG correlates of alertness, neurobehavioral measures and subjective sleepiness, and suggests additional factors may influence the time taken for ABI patients to self-report a change in their symptoms. Some questionnaire items, do not lend themselves to reporting rapid or acute changes in symptoms. For example, the FSS has items capturing impact of fatigue on daily activities, and general statements such as ‘My motivation is lower when I am fatigued.’

Inter-individual variability in response to the light treatment was considerable, as observed by large change score standard deviations, and visual inspection of individual plots. These differences could be associated with a number of factors. Firstly, it was challenging to examine participants’ treatment compliance. A few participants with memory deficits expressed difficulty recalling their treatment adherence. No memory measures were included as the study protocol was already burdensome, but future studies could incorporate such measures to identify whether memory impacts compliance, and consider use of daily reminders, or automated systems that do not require user input. Psychosocial factors also affected some individuals during the study, potentially impacting their response to the therapeutic conditions, including their mood. Two participants had partners who had children during treatment, which likely increased their fatigue and sleep disruption. Other factors cited by participants included changes in work commitments and schedules, and transitioning to retirement between study conditions. In addition, inter-individual variability in treatment response may have been associated with individual differences in light sensitivity, which requires investigation [61]. Injury severity however, was not found to significantly contribute to treatment response.

Whilst the current sample size is comparable to previous light therapy trials in TBI [25–28], and despite some significant treatment effects being observed, the sample size in the current RCT was small and heterogenous in nature, in terms of the nature and severity of injury, and personal factors. Given that the impact of in-home light intervention on fatigue may be smaller than that following exposure to a light-box, larger samples than anticipated are likely to be necessary to detect effect. However, this pilot RCT has provided support for the expansion of this study in a larger clinical trial. Increasing number of stroke participants is certainly warranted to further explore the efficacy of light therapy in this group, and to analyse the role of injury type in facilitating a treatment effect.

## Conclusions

This pilot trial study is the first of its kind to develop and test a home-based light therapy to treat fatigue and sleep difficulties following ABI. It represents a novel adaption of principles emerging within sleep and circadian neuroscience. The study mirrors recent trends within medicine which aim to deliver individually tailored therapies to patients within the context of their daily lifestyle. The results of the study showed that the treatment lighting significantly reduced sleep disturbance and insomnia symptoms, and improved psychomotor vigilance and productive activity. Changes in fatigue, daytime sleepiness, objective sleep measures, depressive symptoms, and participation were non-significant, but most showed medium effect sizes of improvement. These results are encouraging and suggest that a personalized home-based light therapy may offer an effective, safe, low-demand and long-term therapy for alleviating sleep disturbance in individuals with TBI or stroke.

## Supplementary Information

**Additional file 1**: **Supplementary Table 1**. Participant Demographics. **Supplementary Table 2**. Side effects by Study Condition. **Supplementary Table 3**. Mean and standard deviation of melanopic EDI, melanopic DER, photopic illuminance (lux) and correlated color temperature (CCT).

## Data Availability

The datasets used and/or analysed during the current study are available from the corresponding author on reasonable request.

## Abbreviations

ABI: Acquired brain injury
BFI: Brief Fatigue Inventory
CCT: Correlated color temperature
ESS: Epworth Sleepiness Scale
FSS: Fatigue Severity Scale
GCS: Glasgow Coma Scale
HADS: Hospital Anxiety and Depression Scale
ISI: Insomnia Severity Index
LED: Light Emitting Diode
melanopic DER: Melanopic Daylight Equivalent Ratio
melEDI: Melanopic Equivalent Daylight Illuminance
POPS: Participation Objective Participation Subjective
PSQI: Pittsburgh Sleep Quality Index
PTA: Post-traumatic amnesia
PVT: Psychomotor vigilance task
RT: Reaction time
TBI: Traumatic brain injury
WASO: Wake after sleep onset
