# Supplementary material for: Home-based light therapy for fatigue following acquired brain injury: a pilot randomized controlled trial
Source: BMC Neurol. 2021 Jul 5;21:262. doi: 10.1186/s12883-021-02292-8 (PMC8256500; doi:10.1186/s12883-021-02292-8)
Supplement: Supplementary file 1 — Additional file 1: Supplementary Table 1. Participant Demographics. Supplementary Table 2. Side effects by Study Condition. Supplementary Table 3. Mean and standard deviation of melanopic EDI, melanopic DER, photopic illuminance (lux) and correlated color temperature (CCT). [file 12883_2021_2292_MOESM1_ESM.docx]

**Supplementary Materials**

Table 2 measurements were taken with a Colormunki Light Meter (X-Rite, Grand Rapids, MI, USA) using f.luxometer software (f.lux, Los Angeles, CA, USA), at a fixed height (72”) in the horizontal plane.

‘Day’ measurements were taken with day lighting on and exposure to natural light via windows if present. Day measurements therefore represent maximum possible exposure to lighting during daytime. Evening measurements were taken with evening lighting on and window blinds closed, to approximate night time lighting conditions.

A total of 485 measurements were taken across Treatment and Control conditions; 384 were used in the Table 2 calculations. Measurements were excluded from average calculations in the following circumstances: a) no blinds available to block natural light from windows in 'evening' measurements (12.6% of measures); b) if the space was used exclusively during daytime and evening lighting was not fitted, the evening measure was excluded from averages (3.3%) ; c) if the space was used exclusively during evenings and daytime lighting was not fitted, the daytime measure was excluded (2.3%); on a few occasions, a measure was not taken in a room or setting and represent missing data (2.7%).

The number of participant measures included in the average is represented by *N.* Some areas were not present in homes of some participants (e.g. only 10 participants had a study area).

Supplementary Table 1

*Participant Demographics*

| Baseline Variables | Treatment-Placebo (*n* = 16) | | Placebo-Treatment Sequence (*n* = 8) | | Total (*N* = 24) | |
| --- | --- | --- | --- | --- | --- | --- |
|  | *M* | *SD* | *M* | *SD* | *M* | *SD* |
| Age (years) | 43.13 | 10.67 | 46.75 | 13.13 | 44.33 | 11.39 |
| Gender (female) | 7 (44%) |  | 3 (38%) |  | 10 (42%) |  |
| Years of education | 14.53 | 3.12 | 14.13 | 3.04 | 14.50 | 3.19 |
| Occupation  Unemployed  Trade  Professional  Administration  Arts  Other | 4 (25%)  2 (13%)  4 (25%)  3 (19%)  1 (6%)  2 (13%) |  | 2 (25%)  1 (13%)  0 (0%)  4 (50%)  1 (13%)  0 (0%) |  | 6 (25%)  3 (13%)  4 (17%)  7 (29%)  2 (8%)  2 (8%) |  |
| Ethnicity  Australian  New Zealander  S.E. Asian  N.W. European  S.E. European  South American | 10 (63%)  0 (0%)  2 (13%)  2 (13%)  2 (13%)  0 (0%) |  | 6 (75%)  1 (13%)  0 (0%)  0 (0%)  0 (0%)  1 (13%) |  | 16 (67%)  1 (4%)  2 (8%)  2 (8%)  2 (8%)  1 (4%) |  |
| Living circumstances  Independent  Partner  Partner/children  Parents | 4 (25%)  5 (31%)  4 (25%)  3 (19%) |  | 0 (0%)  4 (50%)  2 (25%)  2 (25%) |  | 4 (17%)  9 (38%)  6 (25%)  5 (21%) |  |
| Bed partner  Always  No  Sometimes | 10 (63%)  5 (31%)  1 (6%) |  | 5 (63%)  3 (38%)  0 (0%) |  | 15 (63%)  8 (33%)  1 (4%) |  |

*Notes.* Occupation represents current occupation only (not historical). Occupation and ethnicity categorized using Australian Bureau Statistics (ABS) classifications.

Supplementary Table 2

*Side effects by Study Condition*

|  | Treatment | | | | | | Control | | | | | |
| --- | --- | --- | --- | --- | --- | --- | --- | --- | --- | --- | --- | --- |
|  | Mild | | Moderate | | Severe | | Mild | | Moderate | | Severe | |
|  | N | % | N | % | N | % | N | % | N | % | N | % |
| Headache | 7 | 30 | 4 | 17 | 3 | 13 | 9 | 39 | 3 | 13 | 3 | 13 |
| Eye irritation | 8 | 35 | 2 | 9 | 1 | 4 | 5 | 22 | 4 | 17 | 3 | 9 |
| Vision problems | 7 | 30 | 4 | 17 | 0 | 0 | 4 | 17 | 6 | 26 | 1 | 4 |
| Nausea or vomiting | 2 | 9 | 1 | 4 | 0 | 0 | 6 | 26 | 0 | 0 | 0 | 0 |
| Increased appetite | 2 | 9 | 0 | 0 | 1 | 4 | 3 | 13 | 0 | 0 | 1 | 4 |
| Decreased appetite | 2 | 9 | 0 | 0 | 0 | 0 | 5 | 22 | 2 | 9 | 0 | 0 |
| Abdominal discomfort | 6 | 26 | 3 | 13 | 0 | 0 | 6 | 26 | 2 | 9 | 1 | 4 |
| Tightness in chest | 7 | 30 | 1 | 4 | 0 | 0 | 5 | 22 | 1 | 4 | 0 | 0 |
| Sleep problems | 8 | 35 | 11 | 48 | 0 | 0 | 9 | 39 | 7 | 30 | 4 | 17 |
| Thought, concentration and memory problems | 13 | 57 | 8 | 35 | 1 | 4 | 10 | 43 | 10 | 43 | 1 | 4 |
| Drowsiness | 6 | 26 | 5 | 22 | 2 | 9 | 8 | 35 | 8 | 35 | 1 | 4 |
| Fatigue | 9 | 39 | 11 | 48 | 3 | 13 | 5 | 22 | 13 | 57 | 4 | 17 |
| Dizziness | 9 | 39 | 2 | 9 | 0 | 0 | 8 | 35 | 4 | 17 | 0 | 0 |
| Anxiety | 9 | 39 | 3 | 13 | 2 | 9 | 6 | 26 | 3 | 13 | 4 | 17 |
| Irritability | 14 | 61 | 3 | 13 | 1 | 4 | 7 | 30 | 6 | 26 | 3 | 13 |
| Feeling Depressed | 12 | 52 | 3 | 13 | 1 | 4 | 11 | 48 | 4 | 17 | 2 | 9 |

*Note.* Values represented above are for end-condition assessments only (*N* = 23). Participant 002 not included in above table due to missing data.

Supplementary Table 3

*Mean and standard deviation of melanopic EDI, melanopic DER, photopic illuminance (lux) and correlated color temperature (CCT)*

|  | **Treatment** | | | **Control** | |
| --- | --- | --- | --- | --- | --- |
| **Measurement** | | **Day** | **Evening** | **Day** | **Evening** |
| **Melanopic EDI** | | 349.50 ± 224.52 | 95.84 ± 64.98 | 225.71 ± 142.49 | 138.68 ± 77.21 |
| **Melanopic DER** | | 0.80 ± 0.07 | 0.42 ± 0.06 | 0.56 ± 0.07 | 0.49 ± 0.07 |
| **Photopic lux** | | 440.97 ± 266.70 | 209.77 ± 151.85 | 393.64 ± 216.39 | 272.36 ± 131.46 |
| **CCT** | | 5273 ± 537 | 2865 ± 206 | 3465 ± 436 | 3214 ± 474 |

*Note.* Measures are averaged across all available spaces utilized during the study, as per the approach outlined in the Supplementary Materials.
